# Repeated intracerebral hemorrhage after craniotomy for a distal middle cerebral artery aneurysm: A case report

**DOI:** 10.1097/MD.0000000000029223

**Published:** 2022-05-06

**Authors:** Yan Feng, MingJun Ji, Yufeng Ren, Ziqian Liu, Zhenxue Xin, Liqun Wang

**Affiliations:** a Department of Neurosurgery, The Second Hospital of Hebei Medical University, No. 215 Heping West Road, Shi Jiazhuang, Hebei, China; b Department of Critical Care Medical, Linxi County People's Hospital, Xing Tai, Hebei, China; c Department of Neurosurgery, The Second People's Hospital of Liaocheng, Liaocheng, Shandong, China.

**Keywords:** craniotomy, computer tomography, distal middle cerebral artery aneurysms, digital subtraction angiography, intracerebral hemorrhage, middle cerebral artery

## Abstract

**Rationale:**

Distal middle cerebral artery aneurysms are very rare in the clinic, and craniotomy clipping is the better treatment after diagnosis. However, patients can also have repeated acute intracerebral hemorrhage after craniotomy for aneurysm, which has not been previously reported.

**Patient concerns:**

A 24-year-old male patient was admitted to our hospital with headache, nausea, and vomiting. He was well before, had no family history of cerebrovascular disease or hypertension, and had no history of trauma.

**Diagnoses:**

Computer tomography and digital subtraction angiography of the brain revealed intracranial hematoma and an aneurysm located at the M4 segment of the left middle cerebral artery.

**Interventions:**

The patient underwent 2 surgeries to treat the aneurysm, followed by 2 operations for acute cerebral hemorrhage.

**Outcomes:**

Despite repeated surgical treatments, the patient had a poor prognosis and eventually died of respiratory and circulatory failure after repeated brain bleeding.

**Lessons:**

Briefly, it is of great importance to consider the risk factors of cerebral hemorrhage, and provide individualized treatment and psychological counseling for patients with intracerebral hemorrhage.

## 1 Introduction

Craniocerebral aneurysms are a common cerebrovascular disease in neurosurgery and usually represent the clinical manifestation of spontaneous subarachnoid hemorrhage in clinical practice. Patients admitted to the hospital are surgically treated after diagnosis of the head by computer tomography angiography or digital subtraction angiography (DSA). The most common sites of craniocerebral aneurysms are in the internal carotid artery system, including the posterior communicating artery, anterior communicating artery, anterior cerebral artery, middle cerebral artery (MCA), and ophthalmic artery. According to the literature,^[1]^ middle cerebral artery aneurysms (MCAAs) account for about 18% to 22% of intracranial aneurysms, while distal middle cerebral artery aneurysms (DMCAs) are extremely rare in the clinic, accounting for about 5% of MCAA. Due to the special location of MCAA, they are often accompanied by intracerebral hemorrhage (ICH). Hence, craniotomy clipping is often selected as the main treatment. With the progress of neurovascular intervention techniques and methods, successful cases of endovascular treatment of DMCA have also been reported.^[2,3]^ However, aneurysms located at the M4 segment of MCA at the end of the artery are often accompanied by ICH, limiting treatment options. Therefore, clipping or excision by craniotomy and hematoma removal are usually used to treat these aneurysms.^[4]^ Meanwhile, patients suffering from repeated acute ICH in the affected hemisphere after receiving the craniotomy to treat aneurysms are very rare in the clinic. In this case report, the patient with DMCA was admitted in March 2021 and has suffered 5 cerebral hemorrhages in the affected cerebral hemisphere. The patient was jointly treated by the Linxi County People's Hospital, Liaocheng Second People's Hospital, and The Second Hospital of Hebei Medical University. Overall, this case can provide a reference for later clinical treatments of DMCA patients with repeated acute ICH.

## 2 Case presentation

**Figure 1:**
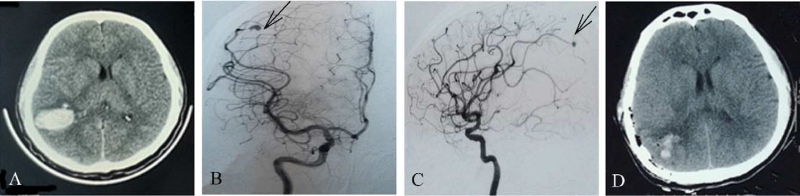
Patient's imaging data during the first ICH. (A) The head CT showed ICH in the right parietal lobe. (B, C) The head DSA (B: anteroposterior view; C: lateral view; black arrow) showed an aneurysm of the distal right middle cerebral artery (M4 segment). (D) The head CT showed that the hematoma was cleared after the first operation (electrocoagulation molding of aneurysm and wrapping). CT = computer tomography, DSA = digital subtraction angiography, ICH = intracerebral hemorrhage.

A 24-year-old male patient visited the Linxi County People's Hospital on March 16, 2021, and presented a sudden headache accompanied by nausea and vomiting. The patient was healthy and had no family history of cerebrovascular disease, hypertension, and infectious diseases. He had no extracranial wounds, the vital signs were stable, and his Glasgow coma scale score was 12. The neurological examination revealed neck resistance (+), motor power of grade IV+/V in the left and right limbs, and left Babinski sign (+). After admission, the head computer tomography (CT) showed ICH in the right parietal lobe (Fig. 1A), and the patient was treated with infusion therapy, including dehydration and reduction of intracranial pressure. The computer tomography angiography revealed a saccular aneurysm in the peripheral cortical segment of the right distal MCA (M4 segment, Fig. 1B, C). On the next day, the patient was transferred to the Second Hospital of Hebei Medical University for surgery, immediately performed to evacuate the ICH. The aneurysm was wrapped after electrocoagulation molding. The operation was smooth. The head CT on the second day after surgery is presented in Fig. 1D. The patient was generally in good condition after surgery and was transferred back to the Linxi County People's Hospital for treatment 2 weeks later.

**Figure 2:**
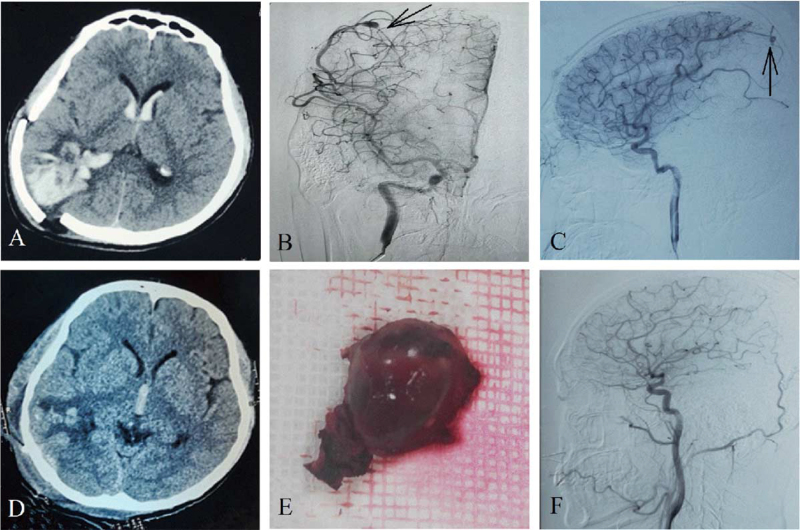
Patient's imaging data during the second ICH. (A) The second head CT showed right parietal hemorrhage into the ventricular system. (B, C) The second head DSA (B: anteroposterior view; C: lateral view; black arrow) showed a cystic aneurysm in its original position. Thus, aneurysm recurrence was considered. (D) Aneurysm resection and removal of ICH were performed during the second operation. The head CT showed that the hematoma was completely removed. (E) Removed aneurysm tissue. (F) In the third head DSA (lateral view) no aneurysms were observed, and there was nothing unusual regarding the course of the intracranial arteries. CT = computer tomography, DSA = digital subtraction angiography, ICH = intracerebral hemorrhage.

On April 6, 2021, the patient presented a sudden headache accompanied by nausea. The head CT showed ICH in the right parietal lobe with intraventricular hematocele (Fig. 2A). The second head DSA was performed to show the original position of the cystic aneurysm. Finally, aneurysm recurrence was considered (Fig. 2B, C). Then, the ICH was removed and the aneurysm was excised in the emergency. The head CT (Fig. 2D) showed that the hematoma was completely cleared after the surgery. The aneurysm removed during surgery is presented in Fig. 2E. After the operation, the patient was treated with infusion therapy to lower the intracranial pressure and anti-infection. The patient was conscious, generally in good condition, and discharged smoothly.

**Figure 3:**
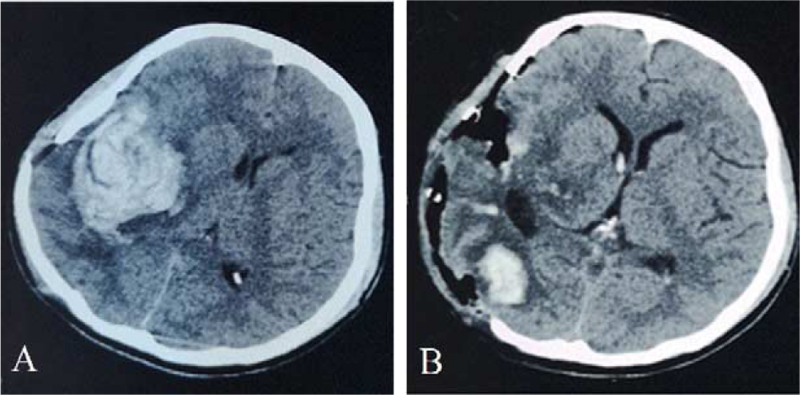
Patient's imaging data during the third ICH. (A) The head CT showed ICH in the right basal ganglia region. (B) Postoperative head CT showed that most of the hematoma was cleared. CT = computer tomography, ICH = intracerebral hemorrhage.

On May 7, 2021, no aneurysms were observed in the head DSA, and nothing unusual was detected regarding the course of the intracranial arteries (Fig. 2F). However, the patient presented sudden unconsciousness and returned to the Linxi County People's Hospital on May 14, 2021, again. The head CT showed ICH in the right basal ganglia region (Fig. 3A), and the patient underwent hematoma removal and decompressive craniectomy (DC) under emergency conditions. The postoperative CT showed (Fig. 3B) that most of the hematoma was cleared with a slight residue in the right occipital lobe. After the operation, the patient received infusion therapy, regained consciousness, and could normally speak, with motor power of grade III in the left limb. Then, the patient was transferred to the Liaocheng Second People's Hospital for rehabilitation on May 21, 2021.

**Figure 4:**
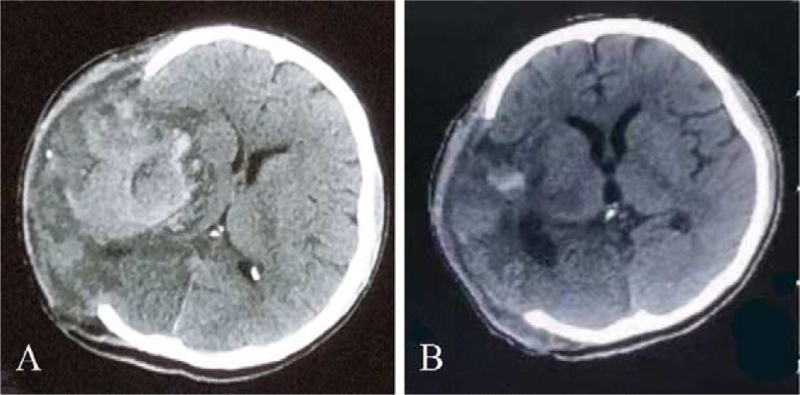
Patient's imaging data during the fourth ICH. (A) The head CT showed ICH in the right basal ganglia region again. (B) Postoperative head CT showed that hematoma was well cleared. CT = computer tomography, ICH = intracerebral hemorrhage.

On May 29, 2021, the patient had another sudden onset of unconsciousness accompanied by vomiting. The head CT showed cerebral hemorrhage in the right basal ganglia region again (Fig. 4A). Thus, right intracranial hematoma removal and internal decompression were performed under emergency conditions, and there was active arterial hemorrhage during the operation. Postoperative head CT (Fig. 4B) showed that the hematoma was cleared. The patient's recovery was satisfactory, with automatic eye-opening and pain localized in the right limb.

**Figure 5:**
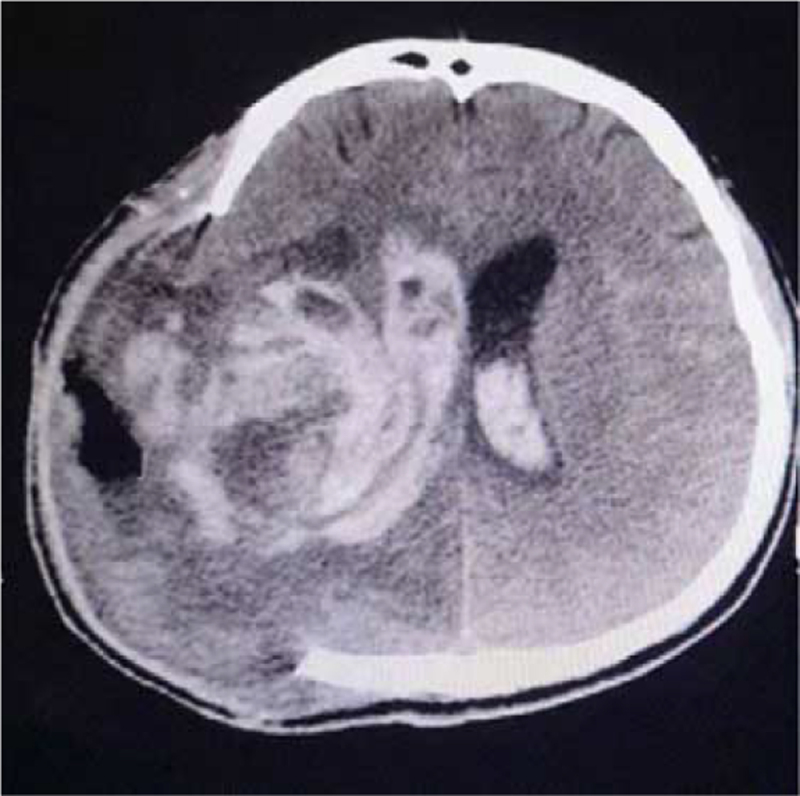
Patient's imaging data during the fifth ICH. The head CT showed right ICH in the same position, which penetrated the bilateral ventricular system. CT = computer tomography, ICH = intracerebral hemorrhage.

Meanwhile, on June 12, 2021, the patient had a sudden onset of blurred consciousness accompanied by vomiting, and the head CT showed right ICH in the same position, which penetrated the bilateral ventricular system (Fig. 5). After obtaining the consent of the patient's family, craniotomy was planned to be performed again. However, during preparation, the patient suddenly suffered respiratory failure and the bilateral pupils dilated, following endotracheal intubation and other rescue treatments. The patient was in critical condition with a poor prognosis and eventually died due to respiratory and circulatory failure.

## 3 Discussion

Since Poppen^[5]^ reported the first distal MCA aneurysm in 1951, several clinical treatments and studies of ruptured distal MCA aneurysms have been developed.^[6,7]^ Generally, a distal MCA aneurysm is an aneurysm occurring at the distal end of the MCA bifurcation (M2 segment and beyond). Aneurysms originating from the distal MCA, or its branches, are rare lesions, presenting an incidence of 2% to 6% among all MCA aneurysms.^[8]^ However, subarachnoid hemorrhage is rarely considered as a clinical manifestation of distal MCA aneurysm since it is usually accompanied by cerebral hemorrhage after rupture. Previously, 34 cases (62%) of ruptured middle cerebral aneurysms accompanied by cerebral hemorrhage were reported among 55 patients,^[9]^ and the incidence of ICH is often related to the aneurysm site. Moreover, the farther away from the MCA, the higher the incidence of ICH.^[10]^ In this case report, the patient was admitted with right parietal cerebral hemorrhage. According to the patient's age and previous medical history, we considered a high possibility of vascular malformation but intracranial aneurysm and moyamoya disease were not discarded. Then, the head DSA confirmed a ruptured aneurysm of the distal right MCA (M4 segment).

For the treatment of patients with distal ruptured aneurysms of MCA accompanied by ICH, most scholars advocate craniotomy clipping and removal of intracranial hematoma to reduce high cranial pressure, and sometimes decompression with bone flap removal. However, distal aneurysms are mostly hidden in the depth of lateral fissure or hematoma. Hence, preoperative planning and vascular reconstruction can provide a favorable guarantee for accurate localization of intraoperative aneurysms. In our case, a temporary occlusion clamp was used to block the artery supplying blood, the aneurysm was wrapped after electrocoagulation molding, and the parent artery was unobstructed. The considerations at the time were: the patient was young, the aneurysm was relatively superficial, and treatment was relatively easy. Placing the aneurysm clamp inside the skull was not considered because the aneurysm cavity was blocked by electrocoagulation and wrapped. Aneurysm wrapping is a procedure in which an artificial dura and autologous muscle fascia are wrapped to strengthen the outer wall of the weak area of the blood vessel wall and reduce the risk of rebleeding. However, this method can not be used to treat aneurysms alone, but only as a reinforcement after aneurysm clipping. Moreover, the patient had recurrent ICH after 2 weeks, and the DSA re-examination also showed aneurysm recurrence. Many factors can affect the recurrence of intracranial aneurysm: incomplete clipping; residual neck or improper intraoperative disposal; location, diameter, and rupture of the aneurysm.^[10,11]^ If we had managed the aneurysm better in the first surgery, either by clipping the aneurysm directly or by removing the aneurysm immediately, the aneurysm might not have recurred.

In clinical practice, nontraumatic spontaneous ICH in young adults between 18 and 45 years is called ICH. There are multiple causes of cerebral hemorrhages in these patients, including hypertension, cerebrovascular malformation, moyamoya disease, and intracranial aneurysm. Other systemic diseases, such as blood and coagulation abnormalities, and special physiological and pathological states, can also play an important role.^[12]^ Additionally, family history, and bad living habits, such as smoking and drinking alcohol, are also important risk factors. Recurrent cerebral hemorrhage occurs when the bleeding stops completely and the brain vessel ruptures again after some time. There are different reports on the incidence of recurrent ICH in the literature. For example, the incidence of recurrent ICH in China is around 4.4% to 21%, and in Asia is about 1.8% to 5.3%.^[13]^ However, the incidence of recurrent ICH in young people has not been previously reported. Postoperative blood pressure instability is a common cause of re-bleeding in patients. The sudden rise of blood pressure accompanied by a sharp increase of cerebral blood flow leads to the re-bleeding of the already occluded blood vessel rupture, comprehending the main factor of postoperative re-bleeding.^[14,15]^ In this case report, the patient recovered smoothly after the second operation (aneurysm resection and removal of intracranial hematoma), and the head DSA re-examination showed no intracranial aneurysm and no abnormality in the intracranial artery. However, the patient suffered the third, fourth, and fifth consecutive ICH, which is very rare in clinical practice. The cause of the first 2 ICH was clear: the aneurysm. On the other hand, the etiology of subsequent ICH remains unclear. The patient had no history of hypertension, no family history of cerebrovascular disease, and denied smoking and drinking. Moreover, no coagulation dysfunction was detected during and after surgery, and the patient's blood pressure was well controlled after surgery. Therefore, the consideration of patients in the special physiological and pathological state after surgery plays an important role. The patient underwent many craniotomies, which can lead to different negative psychological problems in the stress state, which is also a risk factor for recurrent cerebral hemorrhage.

Furthermore, cerebral amyloid angiopathy (CCA) is also an important cause of recurrent cerebral hemorrhage. This is a unique cerebrovascular disease for the elderly and often occurs in small cortical or meningeal blood vessels. Bleeding sites are located in the brain lobe, irregular shape, point flaky or miliary, and, sometimes, bleeding lesions can fuse. The patient's age, bleeding site, and morphology were not consistent with this type of disease. Hence, CCA-related cerebral hemorrhage was not considered. The prognosis of patients with ICH is poor because the recovery of neurological function after hemorrhage is a long process. For patients suffering from recurrent ICH, the shorter the interval between bleeding, the worse the functional status before recurrent hemorrhage, and the higher the risk of death. It has been reported that recurrent ICH has higher disability and fatality rates than single ICH.^[16]^ In this case, the patient died because of the severity of his illness after repeated cerebral hemorrhage, but the cause behind the multiple ICH remains unclear. The primary and secondary ICH were caused by aneurysm rupture, whether the third and subsequent ICH increased the brittleness of brain tissue due to multiple surgical operations. It may also be caused by the rupture of the “micro-aneurysm” formed after the dilation of micro-artery due to the hypoxia of brain tissue after the aseptic inflammatory infiltration in the surgical area. These are only hypotheses and need to be confirmed by subsequent studies.

## 4 Conclusion

Overall, enough attention should be paid to young patients with ICH in clinical practice, because the occurrence of cerebrovascular disease is not only the result of a single factor but the acute onset of multiple factors. Briefly, it is of great importance to consider the risk factors of cerebral hemorrhage and provide individualized treatment and psychological counseling for patients with ICH.

## Author contributions

Approval of final manuscript: all authors.

**Data curation:** Yan Feng, Mingjun Ji.

**Project administration:** Yufeng Ren, Zhenxue Xin, Ziqian Liu.

**Resources:** Yufeng Ren, Zhenxue Xin.

**Software:** Ziqian Liu.

**Writing – original draft:** Yan Feng.

**Writing – review & editing:** Liqun Wang.
